# Laboratory Measurements of Ferric Chloride (FeCl_3_) under Venusian Conditions

**DOI:** 10.1021/acsearthspacechem.5c00132

**Published:** 2025-07-23

**Authors:** Joanna V. Egan, Alexander D. James, John M. C. Plane

**Affiliations:** School of Chemistry, 120986 University of Leeds, Woodhouse Lane, Leeds LS2 9JT, U.K.

**Keywords:** Venus, unknown absorber, ferric chloride, sulfuric acid, absorption, UV−visible spectroscopy

## Abstract

Ferric chloride (FeCl_3_) in sulfuric acid cloud droplets has been proposed to explain the inhomogeneous near-ultraviolet (UV) absorption visible at the Venusian cloud tops. However, the absorption spectrum of FeCl_3_ in concentrated sulfuric acid does not appear to have been measured previously; here we report measurements under appropriate conditions of temperature and H_2_SO_4_/H_2_O solution strengths. The choice of solvent has a significant effect on the measured spectrum. The reaction of FeCl_3_ in aqueous H_2_SO_4_ to form ferric sulfate (Fe_2_(SO_4_)_3_) was shown to be suppressed by adding HCl to the solution (as would occur in the Venusian atmosphere). The FeCl_3_ spectrum in sulfuric acid is shown to be in good agreement with observations of the unknown absorber in Venus’ atmosphere. The presence of Fe_2_(SO_4_)_3_, which absorbs strongly below 320 nm, should be considered when reconstructing Venusian spectra to avoid misattribution of absorption in this spectral region to SO_2_, potentially leading to an overestimation of the SO_2_ cloud top concentrations.

## Graphical abstract

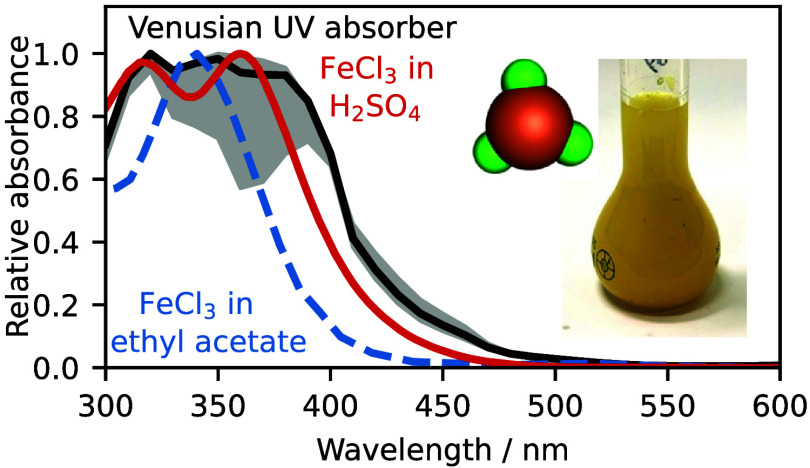

## 1 Introduction

Observation of the Venusian cloud tops at near-ultraviolet (NUV) wavelengths reveals inhomogeneous absorption, the cause of which has remained one of the largest unanswered questions in Venusian research since its original detection in 1927.^1,2^ The Venusian clouds are composed of UV-bright aqueous sulfuric acid (approximately 80 wt %,^3^ though some local variation may occur^4^), which is produced from photochemical oxidation of gas-phase SO_2_ in the presence of H_2_O. Near the cloud tops, two cloud particle size modes have been detected, with modal radii of 200 nm (“mode 1”) and 1.05 μm (“mode 2”).^5^

The cause of the NUV absorption (“the unknown absorber” hereafter) is generally accepted to be located within the upper cloud layer (∼57–70 km^3,5^ ), although the precise altitude range is not known.^6–8^ Models generally cannot distinguish between gaseous and particulate candidates for the unknown absorber, or identify which cloud mode(s) a particulate candidate for the absorber is expected to be in. All retrievals of the shape of the absorption spectrum require the use of models to compare to observations,^6–9^ and as such the retrieved shape of the absorption spectrum is dependent on the assumptions made regarding the altitude profile of the absorber. Probably the best spectrum of the unknown absorber was measured by the MASCS instrument on the MESSENGER spacecraft^9^ during a gravity-assist maneuver at Venus (Figure 1).

**Figure 1:**
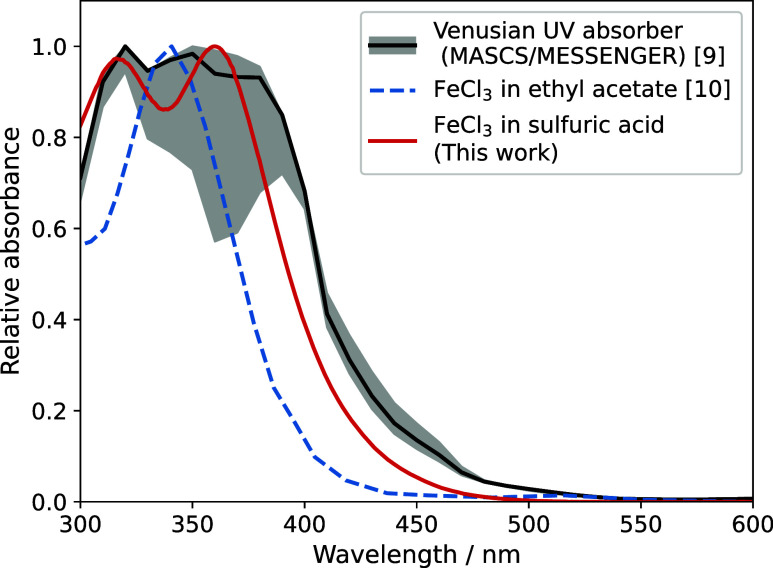
MASCS/MESSENGER spectrum of the unknown absorber (black line and gray uncertainty region)^9^ compared with the FeCl_3_ spectrum measured in ethyl acetate (blue dashed line),^10^ which shows poor agreement, and an example FeCl_3_ spectrum resulting from this work (red).

A large number of species have been proposed as possible causes of the NUV absorption since its detection (e.g., refs 2,9 ). Many candidates are sulfur-based due to the extensive sulfur chemistry on Venus, including OSSO,^11,12^ polysulfur,^13–15^ and polysulfur oxide,^13,14^ though concentrations of these are generally modeled to be too low to account for the observed absorption.^14–17^ Jiang et al.^4^ proposed that iron–sulfur minerals matching the observed absorption spectrum would form in cloud droplets following the uptake and dissociation of ferric chloride (FeCl_3_) and reaction with the sulfuric acid.

FeCl_3_ itself has also been proposed as a possible cause of the absorption: it was included in a list of possible transition metal candidates for the unknown absorber by Kuiper^18^ due to its yellow color. The strongest evidence for FeCl_3_ as the unknown absorber comes from Zasova et al.,^19^ who reported that 1 wt % FeCl_3_ in mode 2 cloud droplets provided a good match to the observed spectrum of Venus. However, they did not publish an absorption spectrum of FeCl_3_, only the predicted spherical (Bond) albedo of the planet. In the absence of an available absorption spectrum of FeCl_3_ in sulfuric acid, recent research has used an alternative spectrum, measured in ethyl acetate, for comparison.^9,10^ The FeCl_3_ spectrum in ethyl acetate bears little similarity to the reported Venusian absorption spectrum (Figure 1). Given that FeCl_3_ can react with ethyl acetate,^20^ the validity of using this spectrum to model FeCl_3_ in the Venusian atmosphere is in doubt. The objective of the present study was therefore to measure a representative FeCl_3_ absorption spectrum for use in atmospheric modeling of Venus, and examine the expected chemical equilibrium of the iron-chloride-sulfur system.

### 1.1 The Fe–Cl–SO_4_ System

Liu et al.^21^ measured UV–vis spectra of FeCl_3_ in ultrasaline solutions of HCl and LiCl. They found five absorbing iron and iron chloride ion complexes (which they identified as Fe^3+^, FeCl^2+^, FeCl_2_^+^, FeCl_3(aq)_, and FeCl_4_^–^) by principal component and “model-free” analysis, and reported that the variety in shape with chloride concentration was due to the dominance of different complexes at different chloride concentrations.

When anhydrous FeCl_3_ is dissolved in concentrated H_2_SO_4_, a complex equilibrium is established. Note that at these concentrations and Venus atmospheric temperatures, H_2_SO_4_ at equilibrium contains a significant concentration of monoprotonated HSO_4_^–^. Considering only the dissociation of the FeCl_3_, and not the reaction to form products, the equilibrium can be written as

2FeCl3+6H2O+3H2SO4⇋2FeCl2++2HSO4−+H2SO4+4H2O+2H3O++2Cl−⇋2FeCl2++2HSO4−+SO42−+2H2O+4H3O++4Cl−⇋2Fe3++3SO42−+2H2O+6H3O++6Cl−

In addition to the proposed high concentrations of FeCl_3_ in the Venusian cloud droplets, gas-phase HCl mixing ratios on the order of 0.1 ppm have been observed near the cloud tops^22–26^ which, by analogy to the Earth’s Junge layer, would result in uptake of HCl into the cloud droplets from the gas phase to saturate the droplets with Cl^–^.^27^ Therefore, in the presence of excess HCl, further chlorinated Fe species are also possible

$${\mathrm{FeCl}}_{3}+\mathrm{HCl}+H_{2}O⇋{\mathrm{FeCl}}_{4}^{-}+H_{3}O^{+}$$

For full consideration of the system, the formation of iron sulfate ions (e.g., FeSO_4_^+^, Fe­(SO_4_)_2_^–^, and FeOH^2+^ ^28^) or minerals (rhomboclase (H_5_O_2_)­Fe­(SO_4_)_2_·3H_2_O, acid ferric sulfate (H_3_O)­Fe­(SO_4_)_2_, and copiapite Fe^2+^Fe_4_^3+^(SO_4_)_6_(OH)_2_·20H_2_O^4^) should also be considered, along with the possible formation of iron chloride sulfates (generic formula FeCl*_n_* (SO_4_)*_m_* ^3–*n*–2*m*^). H_2_O can also form cluster ions with any of the other species. In this study we attempted to identify the end products of these reactions.

We theorize that at high FeCl_3_ concentrations, as proposed Zasova et al.,^19^ a small amount of the FeCl_3_ partially dissociates until the solution is saturated with respect to Cl^–^, reaching an equilibrium in which the rest of the ferric chloride does not fully dissociate. The aqueous ferric chloride (FeCl_3(aq)_) and ferric chloride ions (FeCl*_n_* ^3–*n*^) in the solution may therefore persist for long periods as the rate of reaction is effectively limited by the rate of escape of HCl from the solution into the gas phase above the solution, and from there into the wider atmosphere. At low concentrations of FeCl_3_, the majority of the ferric chloride must dissociate before chloride saturation of the solution occurs, resulting in a rapid reaction to form iron–sulfur ions or minerals.

## 2 Experimental Methods

### 2.1 Scoping Experiments

We performed scoping experiments to measure the real component of the refractive index, to observe the behavior of the solutions over time and to determine what concentration regime would be required to measure absorption coefficients. Initially, high concentrations (∼1 wt %) of anhydrous FeCl_3_ were added to ∼75 wt % sulfuric acid—the concentrations reported by Zasova et al.^19^—and the refractive index measured over 35 days. The sample separated into a liquid phase over a colorless precipitate which was resuspended on mixing. Samples of the pure liquid were taken periodically to measure the absorbance using an ultraviolet–visible (“UV–vis”) Spectrophotometer (See Section 2.2), but all samples saturated the detector in the NUV, and no spectra could be recorded.

When samples were made up with initial FeCl_3_ concentrations decreased by a factor of 1000 to avoid detector saturation, the resulting absorbance spectra measured by UV–vis spectroscopy were consistent with literature spectra identified as ferric sulfate ions (ref 28 and references therein) and with spectra of comparable concentrations of ferric sulfate (Fe_2_(SO_4_)_3_·5H_2_O, Aldrich Chemical Co.) in the same concentrations of sulfuric acid.

### 2.2 UV–Vis Spectroscopy

All experiments use laboratory grade reagents: H_2_SO_4_ (>95%, Fischer Scientific), HCl (∼37%, Fischer Scientific), anhydrous FeCl_3_ powder (MP Biomedicals, stored under nitrogen and in the presence of desiccants), and Fe_2_(SO_4_)_3_·(H_2_O)_5_ powder (Aldrich Chemical Co.).

Absorbance spectra were recorded using an Agilent Cary 100 Ultraviolet–visible Spectrophotometer. Samples were placed in 10 mm optical path length quartz cuvettes and the absorbance measured from 600 to 200 nm with 1 nm resolution. All spectra were recorded relative to a deionized water control with automatic baseline correction. The sides of the cuvettes were cleaned with deionized water and dried with optical lens tissue or acetone when required.

Spectra of FeCl_3_ in H_2_SO_4_ with addition of HCl to drive equilibrium away from ferric sulfate and toward ferric chloride species were measured. To facilitate analysis of the spectra of three component solutions, spectra of FeCl_3_ in HCl and Fe_2_(SO_4_)_3_ in H_2_SO_4_ were also measured.

#### 2.2.1 H_2_SO_4_ + FeCl_3_ + HCl

Two sets of 15 samples (sets “*x*” and “*y*”) were produced containing approximately 77 wt % (12.9 M) and 78 wt % (13.3 M) H_2_SO_4_, respectively. Samples contained varying initial concentrations of HCl (∼0.1, 0.01, and 0.001 M, sample sets 0, 1, and 2, respectively) and FeCl_3_ (9 × 10^–5^–5 × 10^–4^ M, samples B–F). The preparation method is described in Section S1.1 of the Supporting Information (SI), and precise concentrations of each sample are provided in Table S1. Gas bubbles nucleated in the samples during the experiment; this is assumed to be HCl that was released from the sample when Cl^–^ dissociation from FeCl_3_ increased Cl^–^ concentrations above the saturation concentration of the solution (the HCl concentration could not be measured during the experiment). UV–vis spectra of the samples were measured periodically for 99 days (set *x*) and 114 days (set *y*) relative to a deionized water background.

#### 2.2.2 H_2_SO_4_ + Fe_2_(SO_4_)_3_

Twenty-one samples of ∼2.2 × 10^–4^ M Fe_2_(SO_4_)_3_ in 15–98 wt % (1.6–18.3 M) H_2_SO_4_ were measured periodically for 118 days. No significant change in shape with time was seen, and the resulting solution was assumed to be at equilibrium. Precise concentrations of the samples are listed in Table S2 in Section S1.2 of the SI.

#### 2.2.3 FeCl_3_ + HCl and Fe_2_(SO_4_)_3_ + H_2_SO_4_

Samples of 2 × 10^–5^–1.4 × 10^–4^ M FeCl_3_ in 1.5–2.2 M HCl and samples of 5 × 10^–5^–3 × 10^–4^ M Fe from Fe_2_(SO_4_)_3_ in 73–80 wt % (12.1–14.1 M) H_2_SO_4_ were measured and the molar absorptivity of each species calculated. The initial preparation and subsequent dilution of the samples to produce a series of different iron concentrations at a given acid concentration are described in Section S1.3 of the SI. The precise concentrations of FeCl_3_ and HCl and Fe_2_(SO_4_)_3_ and H_2_SO_4_ in the sample sets are provided in Tables S3 and S4.

### 2.3 Nonlinear Fitting

Principal component analysis, as used by Liu et al.,^21^ was unable to separate the species in the H_2_SO_4_/HCl/FeCl_3_ sample spectra. Linear combinations of the spectra reported by Liu et al.^21^ could approximate the measured spectra, but not fully reproduce them. This is assumed to be due to the temperature dependence of the precise shapes of the absorption spectra.

Instead, a fitting algorithm was developed to combine the measured chloride and sulfate spectra. By selecting combinations of the measured spectra, a broader range of complex ion ratios could be modeled. The measured molar absorptivities of the five FeCl_3_/HCl samples, the absorptivity of the Fe_2_(SO_4_)_3_/H_2_SO_4_ samples closest in total iron concentrations to the FeCl_3_/H_2_SO_4_/HCl samples (Sample 77 for sample set *x* and Sample 79 for sample set *y*; see Tables S3 and S4 in the SI), and average absorbance spectra of H_2_SO_4_ and HCl (and a baseline correction if required) were fitted to the FeCl_3_/H_2_SO_4_/HCl absorption spectra.

The algorithm performed fitting with 1 nm resolution from 200–600 nm to a function of the form

$$y\left(\lambda \right)=b+\sum_{i}c_{i}y_{i}\left(\lambda \right)$$

where *c*_*i*_ are the fitting parameters and *y*_*i*_(λ) are the functions being fitted: the average absorbances of HCl and H_2_SO_4_ and molar absorptivities of the Fe sulfate and chloride species, and *b* is a constant baseline. The fitting parameters *c*_*i*_ were constrained to be strictly positive with two exceptions: the baseline and HCl concentration were constrained to be strictly negative. The baseline takes a negative value to allow for zero values where necessary (as all spectra *y*_*i*_(λ) and fitting parameters *c*_*i*_ are positive, except HCl). The HCl is strictly negative to offset the HCl contribution that could not be fully removed in the chloride reference spectra.

When the functions *y*_*i*_(λ) are the absorptivities of each species (multiplied by a path length of 1.0 cm), the fitting parameters are, following the Beer–Lambert law, their concentrations in the mixture. Fitting was performed using python’s nonlinear least-squares minimization (“lmfit”) module.

## 3 Results and Discussion

### 3.1 Scoping Experiments

Ferric chloride can exist as either hydrated (FeCl_3_·6H_2_O, Figure 2a) or anhydrous (Figure 2b) powders, which have very different appearances and behaviors. The hydrate is a yellow crystal, while the anhydrous form is a highly deliquescent black powder, which absorbs ambient water from the air and turns yellow (see the yellow stains on the filter paper in Figure 2b).

**Figure 2:**
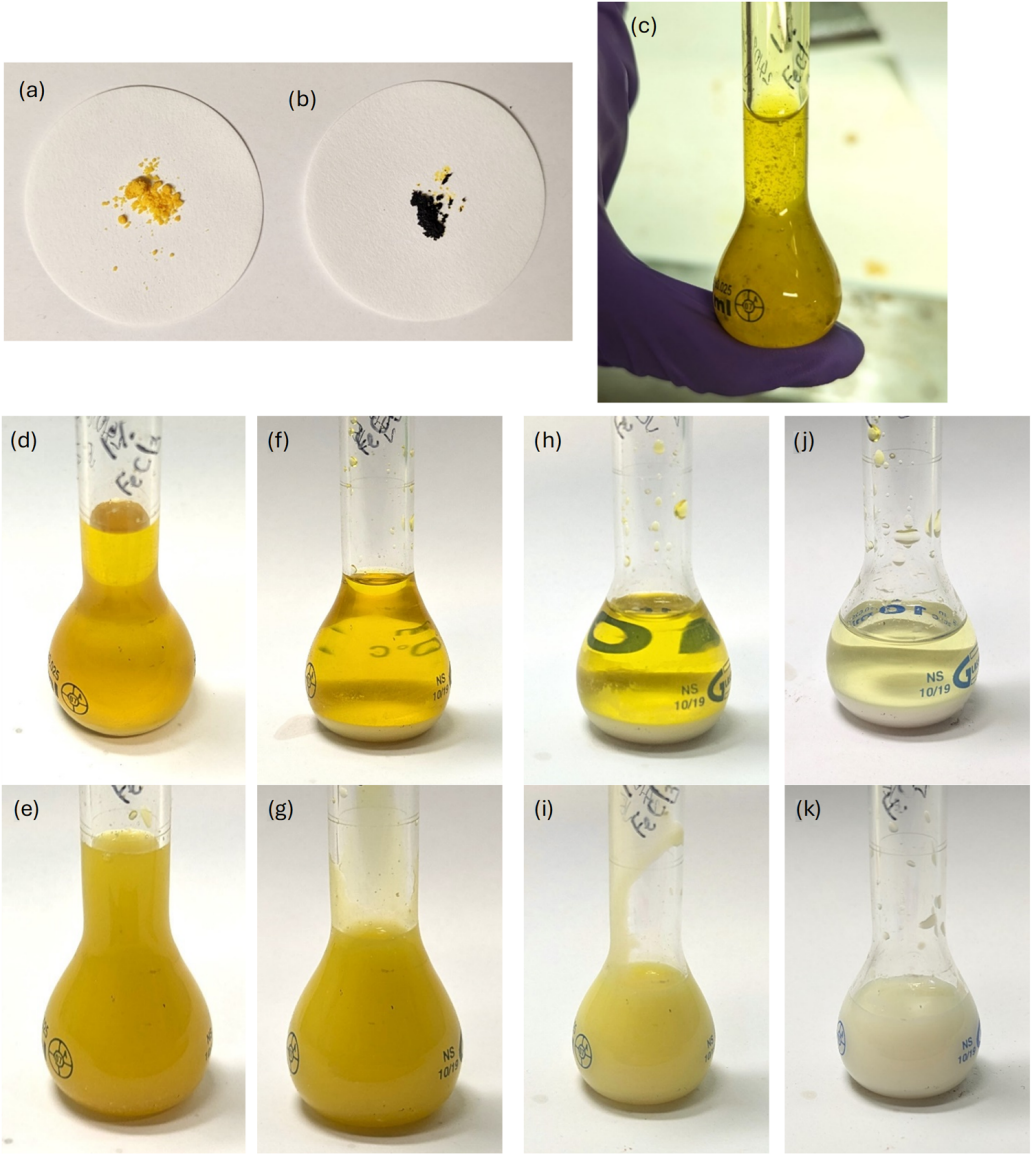
(a) FeCl_3_·6H_2_O powder (Fisons Scientific Equipment), (b) Anhydrous FeCl_3_ powder (MP Biomedicals), (c) 0.95 ± 0.03 wt % anhydrous FeCl_3_ in 76.03 ± 0.02 wt % sulfuric acid (Fischer Chemical, ≥95%) immediately after sample preparation and mixing. The second and third rows show the same sample before inversion (top row) and after inversion (bottom row) after (d, e) 1 day, (f, g) 6 days, (h, i) 13 days, and (j, k) 35 days. Decrease in sample volume over time is due to removal of small volumes for testing.

A solution of ∼1 wt % anhydrous FeCl_3_ in ∼75 wt % sulfuric acid was produced—the concentrations reported by Zasova et al.^19^ The particles initially partially dissolved, producing a strongly yellow-colored liquid with a combination of black and yellow particles suspended (Figure 2c). After 1 day, the solution had formed a clear yellow liquid and a small amount of colorless precipitate (Figure 2d). The sample flask was inverted to mix the solution, producing an opaque yellow liquid (Figure 2e). The solution was then left for 35 days. The volume of the precipitate increased over time (Figure 2f,h,j). Inverting the flask to mix produced opaque liquids throughout (Figure 2g,i,k). Small amounts of the solution were removed frequently (resulting in the volume decrease visible in Figure 2d–k) to measure the real refractive index throughout the experiment. The real refractive index (measured at 589 nm) became consistent with aqueous H_2_SO_4_ after 10 days, while a yellow color was still apparent by eye. After 20 days, the real refractive index was measured to be lower than that of aqueous H_2_SO_4_.

### 3.2 H_2_SO_4_ + FeCl_3_ + HCl

The absorbance spectra of the samples were measured over time. Seven spectra taken after different time periods for each of six samples (FeCl_3_ ∼ 1.7–2.1 × 10^–4^ M, “C” samples—see Table S1 in the SI for precise concentrations) are shown in Figure 3 (spectra were measured more frequently, but only a subset are shown for legibility; the full set of spectra are shown in the SI, Figure S1). The ordinate scale is constant across all plots to aid comparison.

**Figure 3:**
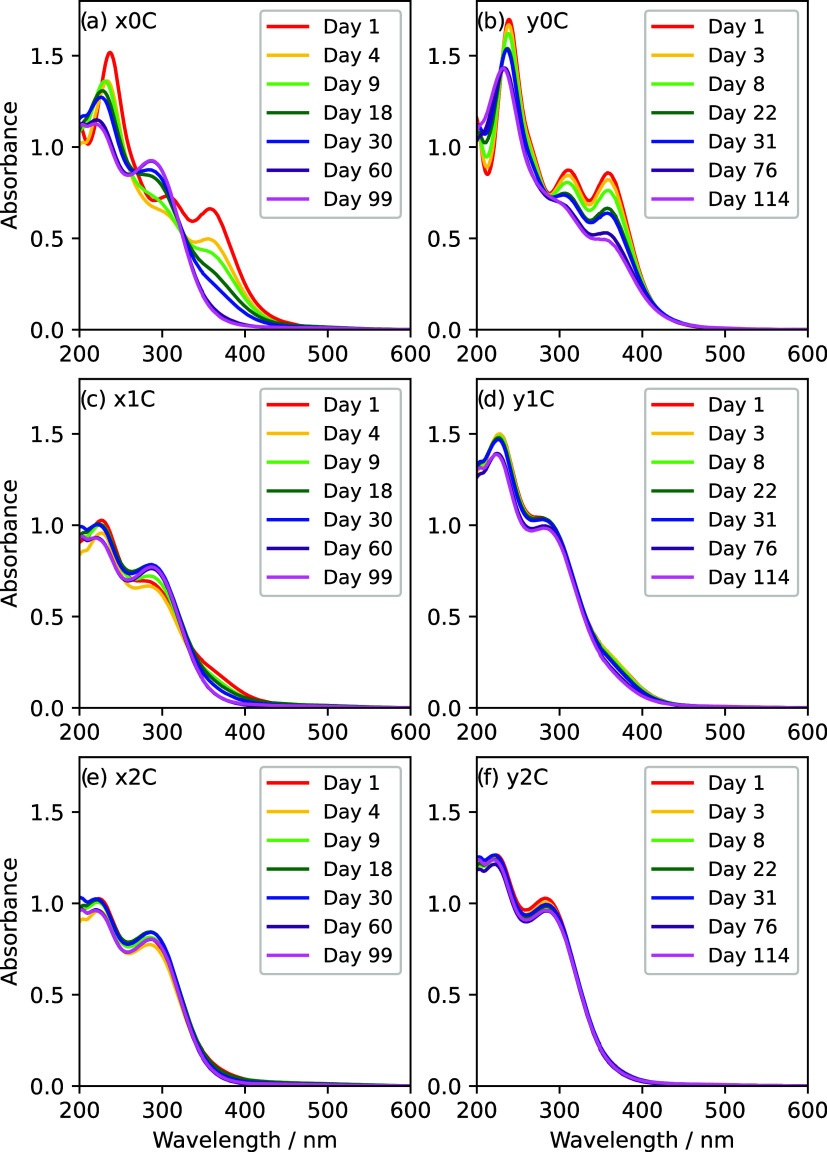
Absorbance spectra measured over time for representative H_2_SO_4_/FeCl_3_/HCl spectra. Samples have initial concentrations of approximately 77 wt % (12.9 M) H_2_SO_4_ for sample set *x* (a, c, e) and 78 wt % (13.3 M) H_2_SO_4_ for sample set *y* (b, d, f), ∼2 × 10^–4^ M FeCl_3_ for all samples, and 9 × 10^–2^ M, 9 × 10^–3^ M, 10 × 10^–4^ M HCl for sample batches 0 (a, b), 1 (c, d), and 2 (e, f), respectively. The reader is referred to Table S1 for precise concentrations for each sample.

At low HCl concentration (Figure 3e,f), the spectra show minimal changes with time and are consistent with Fe_2_(SO_4_)_3_ spectra^28,29^ throughout the course of the experiment. The samples with intermediate HCl concentrations (Figure 3c,d) are consistent with Fe_2_(SO_4_)_3_ at long times, but early spectra show higher absorbance in the 350–400 nm region, which decreases with time. This is more apparent for sample *x*1C (Figure 3c) than *y*1C (Figure 3d), which is not consistent with Fe_2_(SO_4_)_3_ even after 114 days. The high HCl concentration samples (Figure 3a,b) show the clearest change in spectral shape. As for all set *y* samples, the reaction proceeded slowly and had not completed after 114 days, so sample *y*0C was not consistent with Fe_2_(SO_4_)_3_ within the length of the study. Sample *x*0C initially shows a decrease in absorption at all wavelengths above ∼220 nm (days 1–4), followed by a change in shape that sees a decrease in absorption above 350 nm throughout the experiment; an increase near 300 nm from day 4 onward, with a shift in the peak wavelength from 305 to 286 nm, and a decrease in absorbance near 230 nm with a shift of the peak from 237 to 219 nm. The same pattern of behavior with HCl concentration was seen in all samples at different FeCl_3_ concentrations (Figure S1).

By comparison with measured spectra of FeCl_3_ in HCl (Section 3.4) and in high Cl^–^ concentration solutions reported in the literature,^21^ we identify the cause of the higher absorption at the beginning of the experiment as being due to aqueous FeCl_3_ and ferric chloride ions, FeCl*_n_* ^3–*n*^.

### 3.3 H_2_SO_4_ + Fe_2_(SO_4_)_3_

The absorption spectra of samples were measured periodically for 118 days. No significant changes in the absorption spectrum of each sample were observed; also, there was no indication of the formation of solid mineral phases as reported by Jiang et al.^4^ Measurements continued for the same period of time as the H_2_SO_4_/FeCl_3_/HCl samples (Section 3.2) to be confident that no further reactions were taking place after the formation of Fe_2_(SO_4_)_3_ ions. Figure 4 shows the average spectrum of each sample across the experiment.

**Figure 4:**
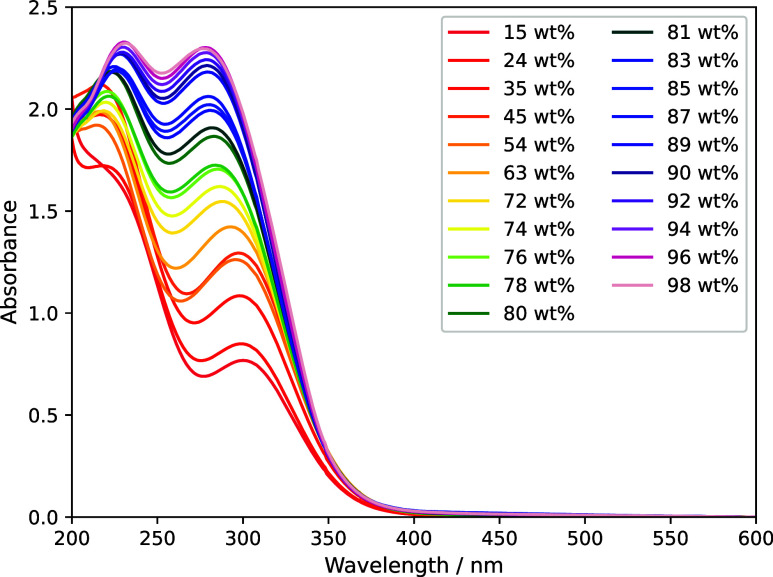
Absorbance measured for ∼2.2 × 10^–4^ M Fe_2_(SO_4_)_3_ in different concentrations of H_2_SO_4_. Samples at 44.7 wt % (darker orange) and 54.7 wt % (lighter orange) do not obey the otherwise universal trend of increasing absorbance in the longer-wavelength peak with increasing H_2_SO_4_ concentration.

With the exception of 44.7 wt % (dark orange) and 54.7 wt % (light orange) H_2_SO_4_, the spectra show a clear trend of increasing peak height near 300 nm and blue shift of the peak as the concentration increases. The difference near 50 wt % is interpreted as the change from a sample dominated near 300 nm by FeSO_4_^+^ (which has an absorptivity maximum of ∼2200 L mol^–1^ cm^–1^ at 300 nm) at low sulfuric acid concentration to a spectrum dominated by Fe­(SO_4_)_2_^–^ (maximum of ∼3000 L mol^–1^ cm^–1^ at 289 nm).^28,29^ Increasing absorption with decreasing wavelength near 200 nm for the 14.7 and 23.8 wt % samples may indicate the presence of small amounts of a third species, possibly FeOH^2+^, which Saunders et al.^28^ reported contributes at low acid concentrations. We conclude that the ferric sulfate spectrum must be measured at the relevant H_2_SO_4_ concentration in order to support interpretation of Fe species in a mixed chloride/sulfate solution.

### 3.4 Iron Chloride and Sulfate Solutions

Each sample spectrum was analyzed relative to the corresponding pure acid. The linearity of sample concentration to absorbance was checked at various wavelengths (SI, Figures S2 and S3) and all sample spectra were shown to be linear in concentration. The molar absorptivities of ferric chloride and ferric sulfate at each acid concentration were calculated and are shown in Figure 5. Greater relative uncertainty near 210 nm in the ferric chloride spectra is due to incomplete removal of HCl signal near 200 nm due to the very high absorbance. The impact on the spectra is minimal; in any case the effect of any remaining HCl spectrum is accounted for in the fitting algorithm (Section 2.3).

**Figure 5:**
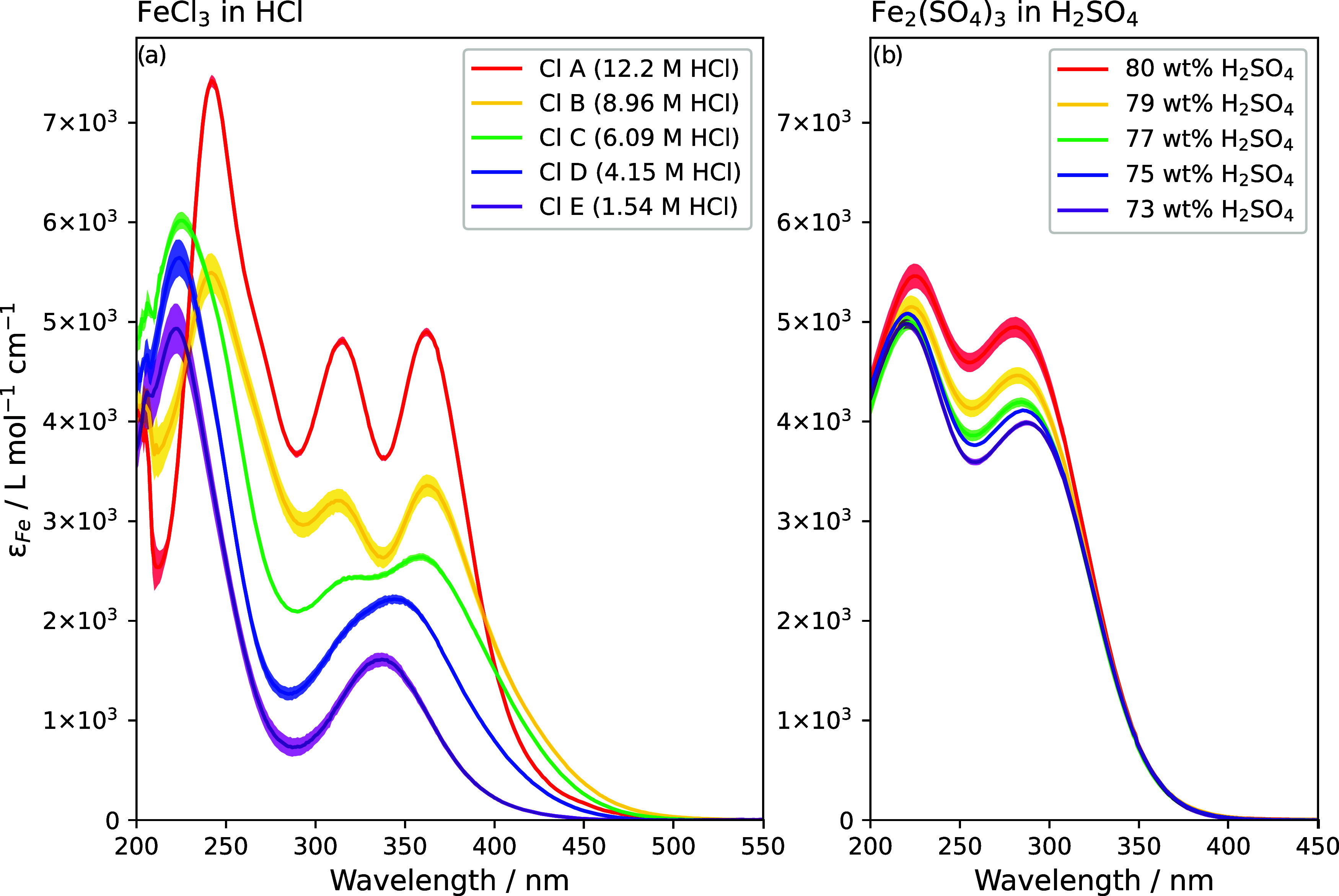
Calculated molar absorptivities of (a) FeCl_3_ in different concentrations of HCl and (b) Fe_2_(SO_4_)_3_ in different concentrations of H_2_SO_4_. Shaded regions show uncertainties resulting from a weighted fit to sample concentrations. Ferric chloride ion spectra are labeled by letter for ease of identification in future figures.

The shapes of the chloride absorptivity spectra vary significantly with HCl concentration. The general trends are similar to those reported by Liu et al.,^21^ who found that the variety in shape with chloride concentration (HCl concentration in this case) was due to the dominance of different complexes at different chloride concentrations. Their spectra and concentrations were found to be temperature dependent, so this experiment is not expected to agree exactly with their results. Nevertheless, there is a qualitative similarity in the change in shape, from a single peak above 300 nm at low chloride concentration (FeCl^2+^), becoming broader and stronger with increasing chloride concentration (FeCl_2_^+^), followed by the formation of two separate peaks (FeCl_3_), which then develop a more pronounced minimum and overall higher absorbance (FeCl_4_^–^) at the highest chloride concentrations.

### 3.5 Fitting Algorithm Results

Fitting was performed using python’s nonlinear least-squares minimization (“lmfit”) module. The reported uncertainties result only from the fitted concentrations of each component, and not from uncertainties in the molar absorptivities shown in Figure 5. The fitting method is not exact, as not all ratios of ions are achievable as weighted sums of their relative concentrations. Nevertheless, this method should provide a reasonable approximation of the concentration of iron chlorides, though individual iron chloride complex ion concentrations are not considered to be quantitatively reliable. The change in total iron chloride concentration over time was used to estimate the rate of conversion of FeCl_3_ to Fe_2_(SO_4_)_3_ ions, which is affected by the concentration of HCl in the solutions.

In this study more than 400 spectra were measured, so Figure 6 shows only a small sample of fit results. Given the necessary simplification of a highly complex system for this fitting, the fitted spectra compare very satisfactorily with the measured spectra. The total iron concentration in each sample (Figure S4) is generally within or just outside the experimental error of the initial concentrations in the sample, with agreement slightly worse in samples dominated by ferric chloride. Due to the known imprecision of the ferric chloride spectra, this is expected.

**Figure 6:**
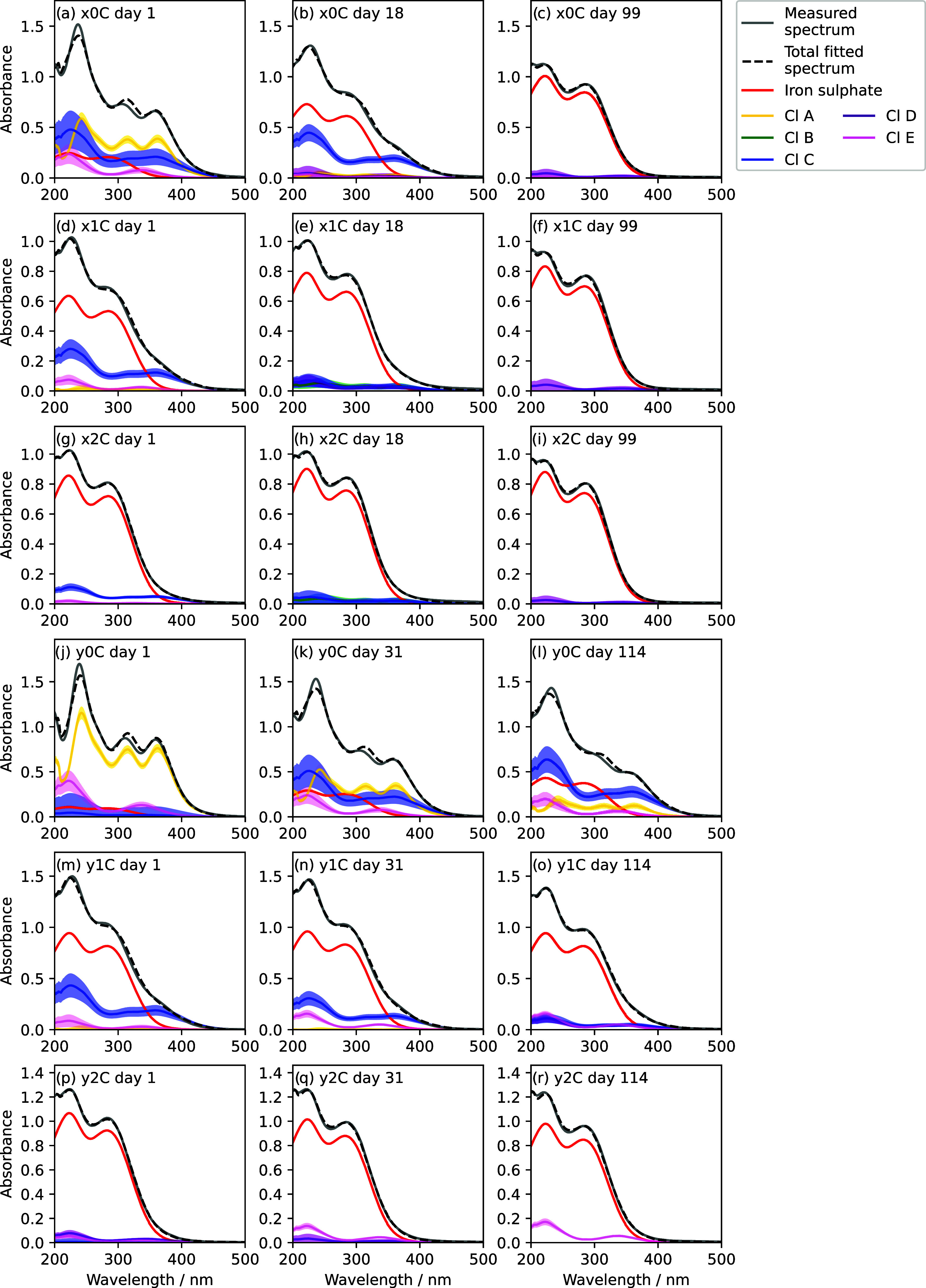
Examples of the fitting algorithm. The measured spectrum (gray line) is fitted by least-squares minimization to produce a total spectrum (black dashed line). Components fitted to the spectrum with concentrations of >10^–8^ M are plotted (different colors) with uncertainties from the fitting algorithm (shaded regions). The reader is referred to Table S1 (SI) for details of each experiment, and to Table S3 and Figure 5 for definition of the iron chloride component names.

The quality of the fit is slightly lower in spectra dominated by chlorides (Figure 6a,j–l), as the fit cannot capture the precise ratio of the ∼240 and ∼305 nm peaks. In spectra with small chloride contributions, i.e., dominated by sulfate (all other subplots), the agreement is clear. In addition to the uncertainty in the shape of the ferric chloride spectra, it is unrealistic to assume that ferric chloride ions would dissociate and react to directly form ferric sulfate ions with no intermediate iron chloride-sulfate complexes. Such complexes would presumably have spectra similar to the pure chlorides and sulfates, though not identical, and would also contribute to the disagreement between measured and fitted total iron.

### 3.6 Rate of Reaction

Using the fitted total iron chloride concentration throughout the experiment, the overall rate of loss of FeCl_3_ was determined. The reaction was found to be first order in all cases, and the rates for experiments using each sample batch were consistent (Figure S5). Note that there was a significant difference between the two batches, with *k* = (5.2 ± 1.6) × 10^–7^ s^–1^ for batch x and (7.1 ± 4.2) × 10^–8^ s^–1^ for batch y at 298 K, which is most likely due to the HCl escaping from the batch stock solution at different rates, thereby changing the Cl^–^ concentration in the liquid that ‘protects’ the FeCl_3_. This could affect not only the rates of specific reactions but also which process is the rate-limiting step in the transformation from chlorides to sulfates, leading to a significant change in the measured rate constant.

## 4 Comparison of the Spectral Shape to Observations

Figure 7 shows the measured absorbance spectrum of FeCl_3_ in samples with a high fitted ferric chloride fraction (Sample *x*0C on day 1, with a fitted total iron chloride to iron sulfate ratio of 82:18%, orange line) and a medium ferric chloride fraction (the same sample on day 18, with a fitted total iron chloride to iron sulfate ratio of 40:60%, yellow line) compared to the absorption spectrum of the unknown absorber estimated from observations and models.^7–9,30^ All spectra have been normalized to a maximum of 1 in the 300–400 nm region for comparison of the shapes of the spectra, with the exception of the day 18 measured spectrum (yellow), which is normalized to 1 at 325 nm for better comparison with the shape of the spectrum reported by Crisp,^30^ based on observations taken during the Pioneer Venus mission.

**Figure 7:**
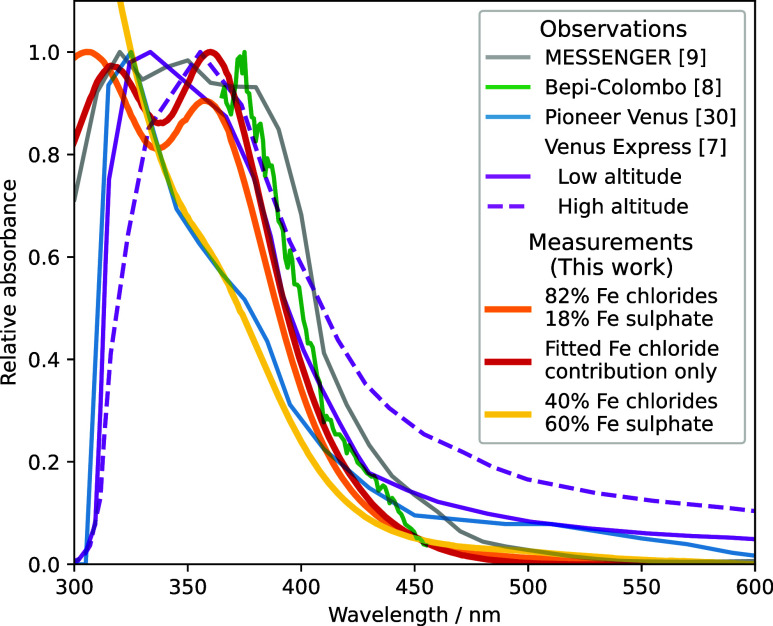
Spectrum of the unknown absorber from models and observations taken during the Pioneer Venus, MESSENGER, Bepi-Colombo, and Venus Express missions^7–9,30^ compared to spectra measured in this work (Sample *x*0C, day 1, orange, and day 18, yellow), and the fitted ferric chloride contribution to the day 1 spectrum (red, also shown in Figure 1). All absorbances have been scaled for comparison of spectral shapes. See Figure 6 for the fitted sulfate/chloride spectra and Table S1 (SI) for initial concentrations of all components.

The shape of the measured spectrum with a high concentration of ferric chloride (Figure 7, orange line) shows better agreement with the observations than prior FeCl_3_ spectra presented in the literature (Figure 1);^9,10^ however, the high absorption near 300 nm, partly due to the presence of ferric sulfate ions (Figure 6a), makes comparison of the shapes difficult. Reflecting the lower temperatures in the upper clouds on Venus than the room temperature of the laboratory and the gas-phase HCl concentrations available to maintain a higher HCl droplet saturation,^27^ the formation of ferric sulfate at early times is likely faster in this experiment than would be expected on Venus.

In addition, the similarity in the absorption regions of the ferric sulfate ions and SO_2_ may have led to misattribution of ferric sulfate absorption to SO_2_, underestimating the absorption due to the unknown absorber (and related species) in the region below 310–320 nm.^7,30^ To account for both of these factors, we reconstruct the absorption spectrum of the ferric chloride alone from the results of the fitting algorithm (Figure 7, red line). This removal of the ferric sulfate improves the agreement between the measured spectra and the observations from Pérez-Hoyos et al.,^9^ using MASCS/MESSNEGER spectra recorded during its 2008 Venus gravity assist, and Lee et al.,^8^ using measurements taken during the 2020 Bepi-Colombo Venus observation period. The inclusion of ferric sulfate in models may also explain reported correlations between the 283 and 365 nm channels of the Akatsuki UV Imager^31^ and the findings by Lee et al.^32^ that the unknown absorber must be modeled to absorb strongly at 283 nm to account for measured phase curves.

The spectrum reported by Crisp,^30^ based on observations recorded during the Pioneer Venus mission, differs significantly from more recent observations and models. The sharp decrease at 310 nm is due to attribution of all absorbance below this value to SO_2_,^30^ leaving a single peak at 325 nm and a “shoulder” in the slope of the absorption at 370 nm. This shape is reminiscent of that of the experimental spectra at longer times (Figure 3a) or intermediate HCl concentrations (Figure 3c) when the spectrum becomes a combination of ferric chloride and ferric sulfate (Figure 6b,d). When a spectrum with comparable concentrations of ferric chloride and ferric sulfate (Sample x0C, measured on day 18, Figure 7, yellow line) is compared to the spectrum reported by Crisp,^30^ there is a clear similarity in the shapes.

The absorption spectrum calculated for the unknown absorber is highly dependent on the altitude profile that is assumed for it. The precise shape may therefore vary. This is most clearly seen in the work of Haus et al.,^7^ who modeled two possible altitude profiles for the unknown absorber, and report very different spectra in each case (Figure 7, solid and dashed purple lines). The agreement of the spectra will therefore be affected by the difference in altitude profile, and the agreement of the measured spectra with observations may be significantly improved by atmospheric modeling. Nevertheless, inspection of Figures 1 and 7 show that the spectra measured in this work provide a much improved fit to the observations, compared with the FeCl_3_ spectrum measured in ethyl acetate.^9,10^ This demonstrates the importance of using the appropriate solvent when measuring the absorption spectrum of ferric chloride for atmospheric modeling purposes.

Good agreement with the spectral shape of the observations indicates that FeCl_3_, if present in sufficient concentrations in the Venusian atmosphere, would largely explain the NUV absorption. However, it is important to note that this does not exclude the existence of other near-UV absorbing species (for example, polysulfur,^13–15^ polysulfur oxide,^13,14^ or iron–sulfur minerals^4^) that may contribute in some wavelength regions.

### 4.1 Comparison to Venusian Conditions

Experiments were carried out at ambient laboratory temperature and pressure. At the expected pressure of ∼0.01–1 bar in the Venusian clouds, the effect of pressure on the reaction within the cloud droplet will be negligible compared to the effects of the liquid phase concentrations of reactants,^33,34^ and this work is therefore expected to be applicable to the full cloud layer without corrections for pressure. Similarly, changes in the shape of the absorption spectrum due to complexation of FeCl_3_ with water ligands at lower acid concentrations are expected to result in only small changes to the absorption spectrum (see SI, Section S3 and Figure S6).

## 5 Conclusions

Despite the adoption of a simple model for what is clearly a complex system, the measured spectra when FeCl_3_ is added to H_2_SO_4_ with HCl can be reproduced satisfactorily with a combination of FeCl_3_ and Fe_2_(SO_4_)_3_ spectra. The limitations of the model—such as the lack of single-component chloride spectra for fitting and the assumption of direct conversion from pure ferric chloride complexes to pure ferric sulfate complexes—undoubtably contribute to uncertainties in the concentrations of the different components. This study shows that the HCl concentration in the solution has a significant effect on the rate of loss of FeCl_3_.

The resulting spectra when FeCl_3_ is added to H_2_SO_4_ solutions are much more similar to observations of the unknown absorber than previous FeCl_3_ spectra which were measured in ethyl acetate (Figure 1).^9,10^ The greater applicability of FeCl_3_ spectra measured in sulfuric acid is self-evident, and these spectra should be used to model FeCl_3_ absorption in the Venusian atmosphere. The exact shape of the absorption spectrum predicted from observations is highly dependent on the assumed altitude profile of the absorber. While the comparison of the measured spectrum to predicted spectra of the unknown absorber are an important first step, and clearly demonstrate that FeCl_3_ is a potential candidate for the unknown absorber, the altitude profile, possible formation pathways, lifetime, and transport through the atmosphere must all be considered through extensive atmospheric modeling to be able to confidently identify FeCl_3_ as the absorber.

The reaction of FeCl_3_ to form Fe_2_(SO_4_)_3_ has been known since FeCl_3_ was proposed as the cause for the absorption.^19^ However, modeling tends to define the wavelength range of the unknown absorber as only that over which absorption cannot be explained by other species, such as SO_2_. Fe_2_(SO_4_)_3_, if present, absorbs in a very similar region in the UV to SO_2_, so the automatic assumption that all absorbance below ∼320 nm is due to SO_2_ may distort the predicted spectral shape of the unknown absorber. When the modeled concentration of Fe_2_(SO_4_)_3_ is removed from the broadly FeCl_3_-dominated spectra measured at early times in this work, the agreement of the measured spectra with the observations improved. If absorption below 320 nm is being misattributed to SO_2_ rather than ferric sulfate, correlations between observations at 283 and 365 nm^31,32^ would be readily explained.

The measured spectrum of the unknown absorber has been predicted very differently over time. This can be seen clearly in Figure 7, with the marked difference between the modeled absorption from the Pioneer Venus campaign^30^ compared to more recent observations^7–9^ clearly apparent. In fact, the general shapes of the earlier and more recent absorption spectra can be reproduced with spectra measured in the present study, if the ratio of FeCl_3_ and Fe_2_(SO_4_)_3_ are different and some absorption below 320 nm has been misattributed to SO_2_, rather than Fe_2_(SO_4_)_3_.

## Supplementary Material



## Data Availability

Data for all figures are available at 10.5281/zenodo.15364165.

## Notes

Section S1: Detailed sample preparation methods and tables of concentrations of all samples; Section S2: Additional figures showing measured absorbance spectra of all H_2_SO_4_ + HCl + FeCl_3_ samples (Figure S1); demonstration of linearity of ferric sulfate and ferric chloride spectra with concentration (Figures S2 and S3); fitted iron chloride, iron sulfate, and total iron concentrations in all H_2_SO_4_ + HCl + FeCl_3_ samples over time (Figure S4); Pseudo-1st order rate constants for all measurable samples (Figure S5); Section S3: Explanation of theoretical absorption spectrum calculations and example predicted spectra in concentrated sulfuric acid and water (Figure S6) (PDF)
