# Supplementary material for: Laboratory Measurements of Ferric Chloride (FeCl3) under Venusian Conditions
Source: ACS Earth Space Chem. 2025 Jul 23;9(8):2127–36. doi: 10.1021/acsearthspacechem.5c00132 (PMC12376184; doi:10.1021/acsearthspacechem.5c00132)
Supplement: Supplementary file 1 [file sp5c00132_si_001.pdf]

Laboratory measurements of ferric chloride ($FeCl_3$) under Venusian conditions

Joanna V. Egan; Alexander D. James; and John M. C. Plane**

School of Chemistry, University of Leeds, Woodhouse Lane, Leeds, LS2 9JT, UK

J.V.Egan@leeds.ac.uk

J.M.C.Plane@leeds.ac.uk

S1. Sample preparation

Solutions were made up by mass. The masses reported in the methods are representative examples. The true mass used was measured at every step in the sample preparation and that measured mass was used to calculate the exact concentrations of samples reported in Tables and Figures. Addition of sulfuric acid was carried out with samples submerged in an ice bath to remove excess heat produced by dilution of the acid.

S1.1 H_2SO_4 + $FeCl_3$ + HCl

Stock solutions of H_2SO_4, HCl, and HCl and $FeCl_3$ were prepared and combined to produce a range of different concentrations for the samples. 500 ml of ~75 wt% sulfuric acid was made up in volumetric flasks with deionized water and concentrated H_2SO_4. The masses of the samples were recorded between addition of reagents to avoid uncertainty in volumetric flasks due to the change in temperature of the solutions through latent heat and use of the ice bath. Three concentrations of HCl were made up by serial dilution to produce concentrations of 12.00(16) M (concentrated HCl), 1.204(16) M, and 0.1199(16) M HCl. 0.096 ± 0.001 g of anhydrous $FeCl_3$ was added to each of three volumetric flasks and made up to 10.00 ± 0.05 ml

with each of the stock HCl solutions. The ratio of HCl and HCl/$FeCl_3$ was varied to produce a range of concentrations of $FeCl_3$ in the same concentrations of HCl.

For each of the 15 samples in each set, a 25.00 ± 0.04 ml volumetric flask was partially filled with stock sulfuric acid and placed in an ice bath. 2 ml of the corresponding sample HCl/$FeCl_3$ solution was added, and then the solutions were topped up with H_2SO_4 as needed. The exact masses were recorded at each step and used to calculate the sample concentrations. Two sets of samples were produced, labelled “x” and “y” to distinguish them. In the first set (x), all steps of sample preparation were preformed immediately after one another. For the second set (y), the HCl/$FeCl_3$ samples were left overnight (~16 hours) to ensure they were fully mixed before final sample preparation by addition of HCl/$FeCl_3$ to the sulfuric acid. The concentrations of the samples are listed in Table S1.

Table S1. Concentrations of H_2SO_4, HCl, and $FeCl_3$ in measured samples. The table defines alphanumeric labels for the samples that are used throughout this work.

Set	Sample	H_2SO_4 concentration		HCl / M	$FeCl_3$ / × 10^{-4} M
		/ M	/ wt %		
x	0B	12.85(8)	76.5(5)	9.452(9) × 10^{-2}	0.863(14)
	0C	12.84(8)	76.5(5)	9.334(9) × 10^{-2}	1.72(3)
	0D*	12.87(8)	76.6(5)	9.158(9) × 10^{-2}	2.49(4)
	0E	12.76(8)	76.5(5)	7.99(8) × 10^{-2}	2.85(5)
	0F	12.90(8)	76.6(5)	9.177(13) × 10^{-2}	4.18(7)
	1B	12.91(8)	76.7(5)	10.250(11) × 10^{-3}	1.43(2)
	1C	12.88(9)	76.7(5)	9.2(3) × 10^{-3}	1.77(7)
	1D*	12.91(8)	76.7(5)	10.065(11) × 10^{-3}	2.97(5)
	1E	12.80(8)	76.9(5)	7.922(10) × 10^{-3}	2.79(5)
	1F	12.87(8)	76.6(5)	10.351(16) × 10^{-3}	4.63(8)
	2B	13.02(8)	76.7(5)	9.96(12) × 10^{-4}	1.33(2)

	2C	13.07(8)	76.5(5)	$10.26(12) \times 10^{-4}$	2.12(3)
	2D*	12.94(8)	76.6(5)	$10.94(13) \times 10^{-4}$	3.28(5)
	2E	12.99(8)	76.8(5)	$8.60(11) \times 10^{-4}$	3.10(5)
	2F	13.10(8)	77.1(5)	$9.67(16) \times 10^{-4}$	4.31(7)
y	0B	13.26(2)	78.12(2)	$9.43(11) \times 10^{-2}$	0.918(10)
	0C	13.36(2)	78.17(2)	$9.52(9) \times 10^{-2}$	1.92(2)
	0D	13.40(2)	78.12(2)	$9.62(10) \times 10^{-2}$	2.88(3)
	0E	13.34(2)	78.161(19)	$9.28(11) \times 10^{-2}$	3.71(3)
	0F	13.48(2)	78.176(18)	$9.37(13) \times 10^{-2}$	4.65(5)
	1B	13.42(2)	78.218(19)	$9.57(11) \times 10^{-3}$	0.945(10)
	1C	13.27(2)	78.049(18)	$9.62(10) \times 10^{-3}$	1.86(2)
	1D	13.36(2)	78.184(15)	$9.37(9) \times 10^{-3}$	2.73(3)
	1E	13.36(2)	78.145(18)	$9.68(11) \times 10^{-3}$	3.78(4)
	1F	13.31(2)	77.976(16)	$10.31(14) \times 10^{-3}$	5.02(5)
	2B	13.31(2)	78.163(18)	$9.58(11) \times 10^{-4}$	0.955(11)
	2C	13.24(2)	77.939(19)	$9.83(10) \times 10^{-4}$	1.90(2)
	2D	13.31(2)	78.080(18)	$9.62(10) \times 10^{-4}$	2.82(3)
	2E	13.42(2)	78.16(2)	$10.22(12) \times 10^{-4}$	4.06(4)
	2F	13.17(2)	78.07(2)	$9.81(13) \times 10^{-4}$	4.79(5)

*D samples from set x showed systematic errors in the initial concentration $FeCl_3$, but the measured concentrations appear reliable across the experiment, see Figure S4.

Bubbles of gas nucleated in the samples on addition of the $HCl/FeCl_3$ solutions to the sulfuric acid and persisted periodically for several days. These are presumed to be HCl which was above its saturation concentration in the sulfuric acid. The HCl concentrations reported are the initial concentrations in the sample, which will have decreased during the experiment. The Cl^- concentration could not be measured, but was assumed to be saturated during the experiment.

S1.2 H_2SO_4 + $Fe_2(SO_4)_3$

21 samples of the same concentration of $Fe_2(SO_4)_3$ in different concentrations of sulfuric acid were measured. No significant change in shape with time was seen, and the resulting solution was assumed to be at equilibrium. Concentrations of the samples are listed in Table S2.

Table S2. Concentrations of $Fe_2(SO_4)_3$ and H_2SO_4 in measured samples.

Sample name	H_2SO_4 concentration		$Fe_2(SO_4)_3$ / $\times10^{-4}$ M
	/ wt%	/ M	
15	14.66(2)	1.653(9)	2.22(6)
24	23.78(2)	2.809(14)	2.16(5)
35	35.321(19)	4.54(2)	2.24(6)
45	44.745(18)	6.10(3)	2.27(6)
54	54.113(17)	7.85(4)	2.24(6)
63	63.289(16)	9.80(5)	2.22(6)
72	72.033(6)	11.844(19)	2.22(5)
74	74.181(6)	12.36(2)	2.24(5)
76	76.073(6)	12.90(2)	2.25(5)
78	77.882(6)	13.35(2)	2.19(5)
80	79.474(6)	13.68(2)	2.29(6)
81	81.285(6)	14.16(2)	2.25(6)
83	83.228(6)	14.79(2)	2.26(6)
85	84.997(6)	15.26(2)	2.24(5)
87	86.804(6)	15.73(3)	2.25(5)
89	88.612(6)	16.21(3)	2.27(6)
90	90.433(6)	16.71(3)	2.25(6)
92	92.305(6)	17.18(3)	2.22(5)
94	94.189(6)	17.61(3)	2.24(5)
96	96.060(6)	18.03(3)	2.27(6)

98	97.633(6)	18.29(3)	2.22(5)

S1.3 $FeCl_3$ + HCl and $Fe_2(SO_4)_3$ + H_2SO_4

Five concentrations of H_2SO_4 stock acids and 6 concentrations of HCl were produced. Stock solutions of the respective iron species were made by adding 0.016 ± 0.001 g $FeCl_3$ to concentrated HCl and 0.034 ± 0.003 g $Fe_2(SO_4)_3.5H_2O$ to ~75 wt% sulfuric acid. Samples were then made by adding ~1 g of iron solution to 10.00 ± 0.05 ml volumetric flasks, which were then made up to volume with the respective acids. The initial ("Undiluted") concentrations of the $FeCl_3$/HCl and $Fe_2(SO_4)_3$/H_2SO_4 solutions are shown in Tables S3 and S4 respectively. To produce diluted samples for the calculation of molar absorptivity of each of the species, the mass of the sample was measured, then ~3 ml removed to fill an optical cell for UV-Vis spectroscopy. The mass was measured again to calculate the exact mass (and therefore volume) removed. The sample from the cell was discarded after measurement, and the volumetric flask filled back to 10 ml with the corresponding acid for that sample. The mass was measured again, and the flask inverted repeatedly to mix the sample. Another ~3 ml was removed for measurement and the procedure repeated to produce a series of decreasing concentrations of iron species at the same concentration of acid.

Table S3. Measured concentrations of $FeCl_3$ in different concentrations of HCl.

Sample	HCl concentration / M	$FeCl_3$ / × 10^{-5} M				
		Undiluted	Dilution 1	Dilution 2	Dilution 3	Dilution 4
A	12.20(6)	10.7(1.0)	7.0(6)	4.6(4)	3.0(3)	1.9(2)
B	8.96(4)	12.8(1.1)	8.5(8)	5.5(5)	3.7(3)	2.4(2)
C	6.09(3)	14.3(1.3)	9.6(9)	6.4(6)	4.2(4)	2.7(2)
D	4.15(2)	11.6(1.0)	7.8(7)	5.2(5)	3.4(3)	2.2(2)
E	1.543(8)	13.0(1.2)	9.0(8)	6.1(5)	4.1(4)	2.8(3)

Table S4. Measured concentrations of Fe from $Fe_2(SO_4)_3$ in different concentrations of H_2SO_4.

Sample	H_2SO_4 concentration		Fe / × 10^{-4} M					
	/ M	/ wt%	Dilution number					
			0	1	2	3	4	5
73	12.14(6)	73.095(12)	3.4(3)	2.25(18)	1.57(13)	1.10(9)	0.76(6)	0.55(4)
75	12.54(6)	75.005(15)	3.4(3)	2.38(19)	1.67(14)	1.19(10)	0.81(7)	0.56(5)
77	13.11(7)	76.824(12)	3.4(3)	2.31(19)	1.67(14)	1.17(10)	0.80(7)	0.55(5)
79	13.51(7)	78.625(13)	3.3(3)	2.37(19)	1.71(14)	1.23(10)	0.86(7)	0.58(5)
80	14.07(7)	80.327(12)	3.4(3)	2.28(18)	1.56(13)	1.08(9)	0.75(6)	0.54(4)

S2. Additional figures

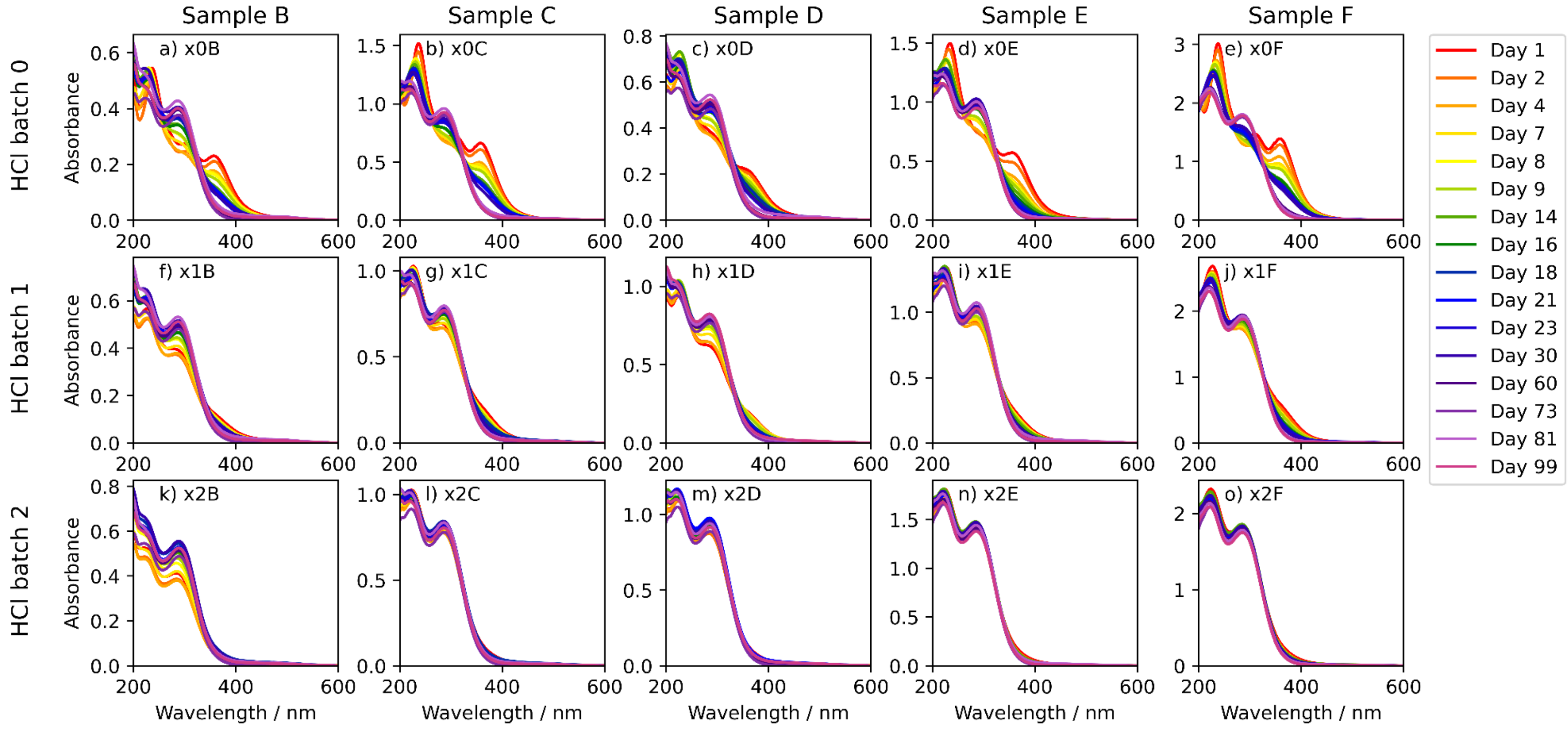

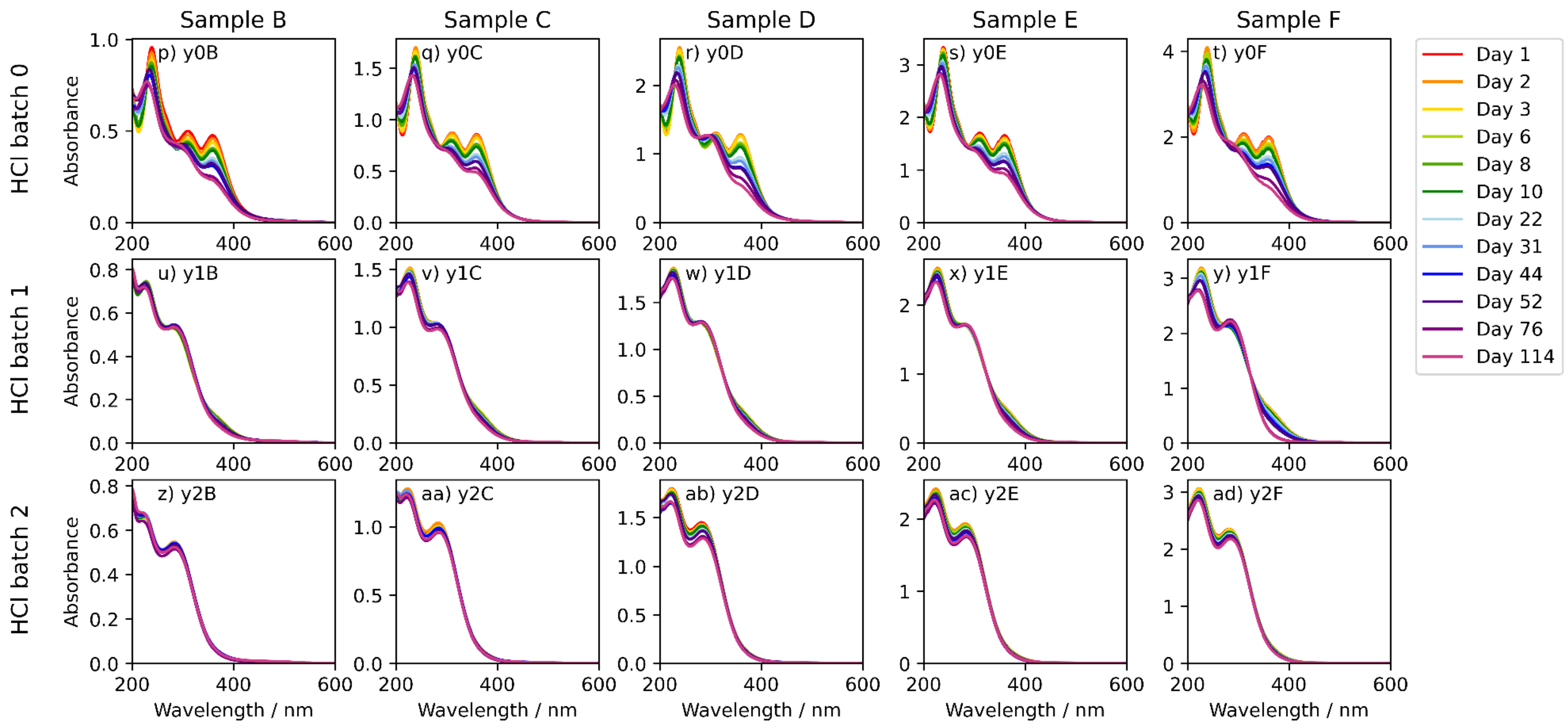

Figure S1. Measured absorbance spectra of all 30 samples of H_2SO_4/HCl/$FeCl_3$ from the x sample set (top three rows) and y sample set (bottom 3 rows). xD samples show a systematic error in the $FeCl_3$ concentration, producing lower than expected absorbance at all times. Within each batch, the same trends can be seen as were outlined in the main paper: HCl batch 2 samples are broadly consistent with ferric sulphate ions at all times and show minimal change, batch 1 samples show a decrease above 350 nm (a decrease near 220 and increase near 300 nm can also be seen in higher $FeCl_3$ concentration samples), and batch 0 samples are show a distinct shape change from chloride-dominated spectra to sulphate dominated spectra at long times. H_2SO_4, and initial $FeCl_3$ and HCl concentrations for each sample are available in Table S1.

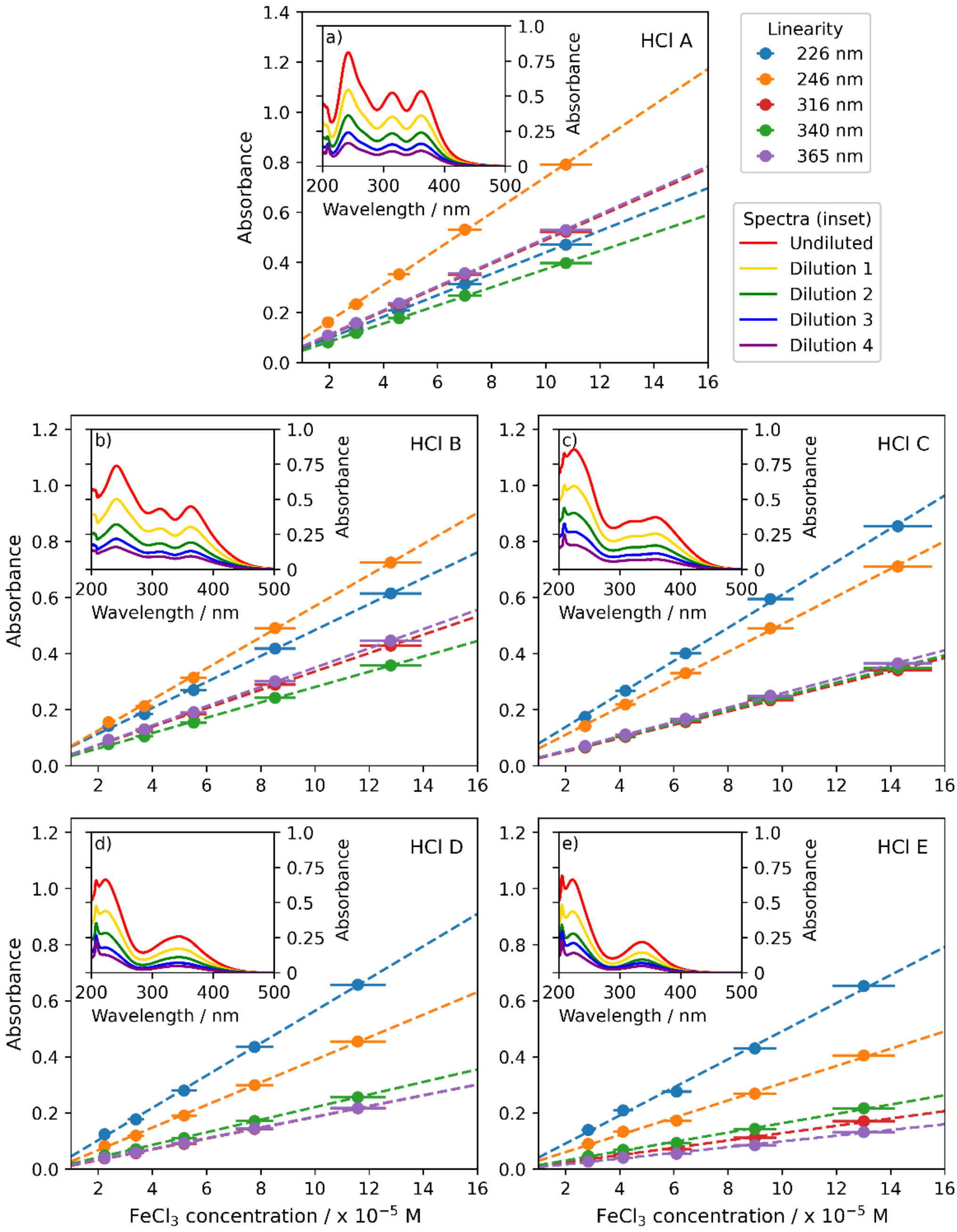

Figure S2. All sample absorbances are linear in concentration at all wavelengths. Inset: the measured spectra. Increasing absorbance near 200 nm and spikes below 215 nm are due to incomplete removal of the HCl sample (which saturated the detector at all concentrations). These features are not dependent on the iron concentration and so are mostly removed by the calculation of molar absorptivity. An HCl spectrum is also included in the fitting to account for any remaining HCl signal in the spectra used. The reader is referred to Table S3 for interpretation of sample names and corresponding concentrations of HCl and $FeCl_3$.

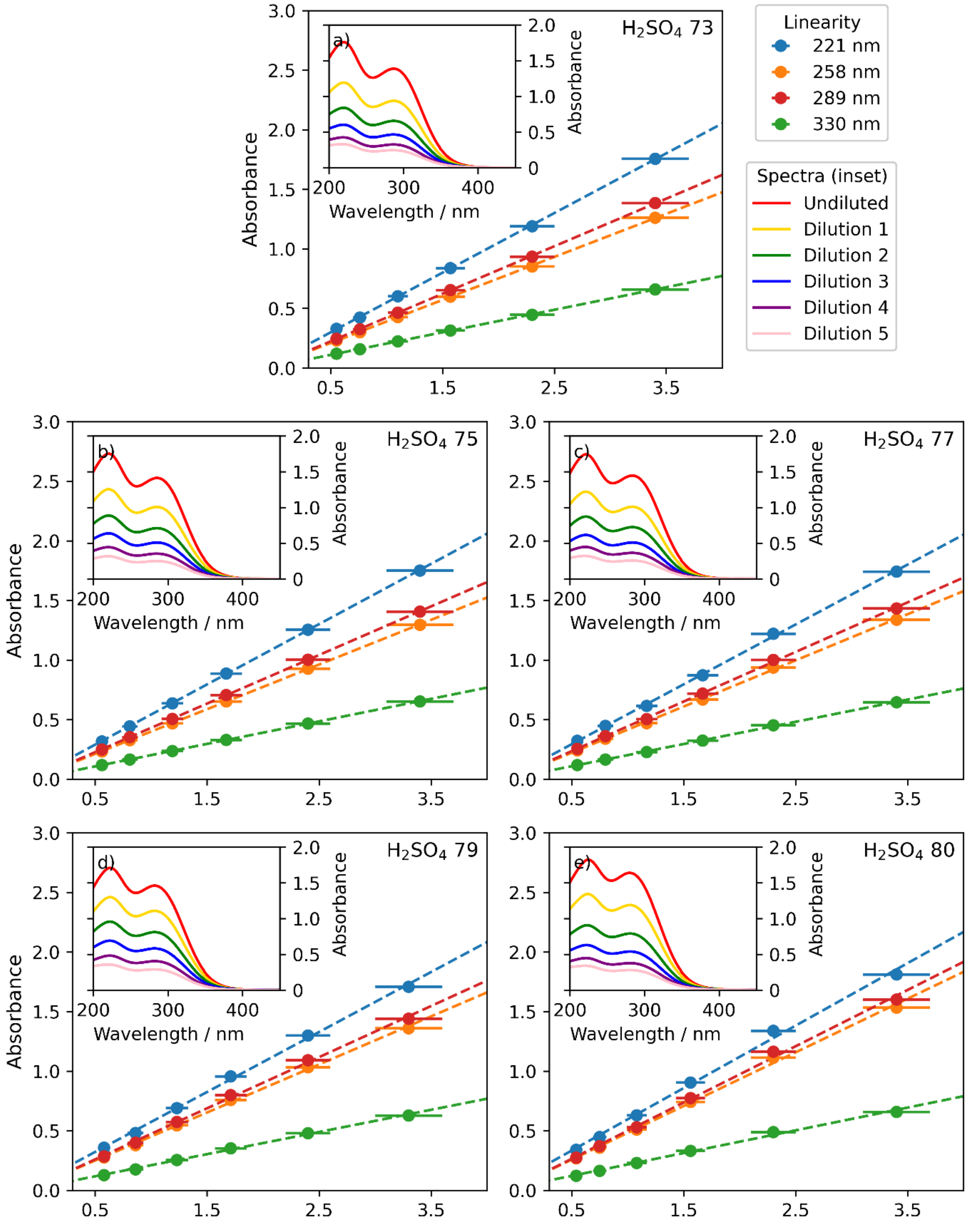

Figure S3: All sample absorbances are linear in concentration at all wavelengths. Inset: The measured spectra with H_2SO_4 background removed. The change in the ratio of the peak heights with sulphuric acid concentration is less obvious with all spectra on separate axes than in Figures 4 and 5b of the main paper. The reader is referred to Table S4 for interpretation of sample names and precise concentrations of H_2SO_4 and $Fe_2(SO_4)_3$.

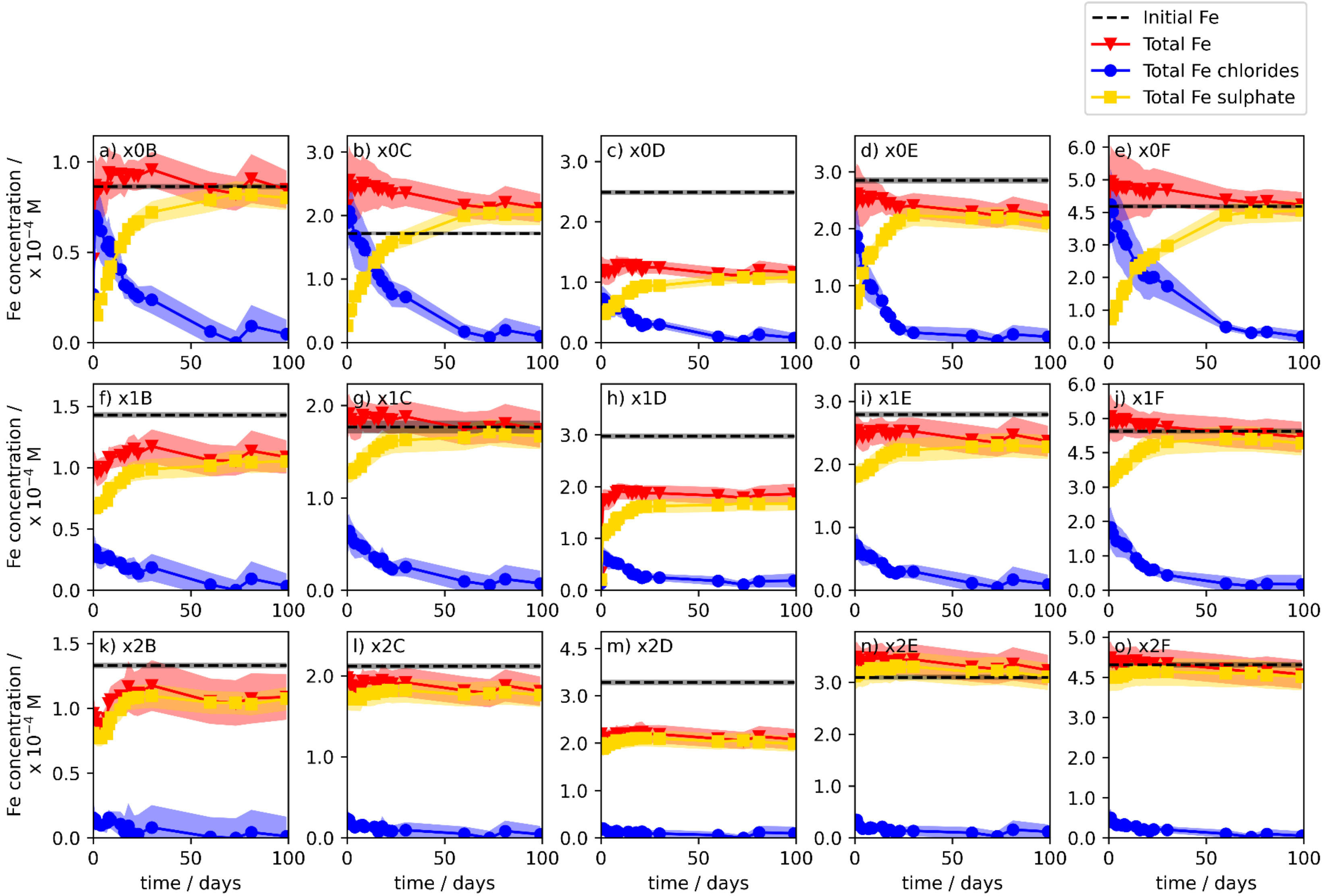

Initial Fe
Total Fe
Total Fe chlorides
Total Fe sulphate
a) x0B
b) x0C
c) x0D
d) x0E
e) x0F
f) x1B
g) x1C
h) x1D
i) x1E
j) x1F
k) x2B
l) x2C
m) x2D
n) x2E
o) x2F
Fe concentration / x 10⁻⁴ M
time / days

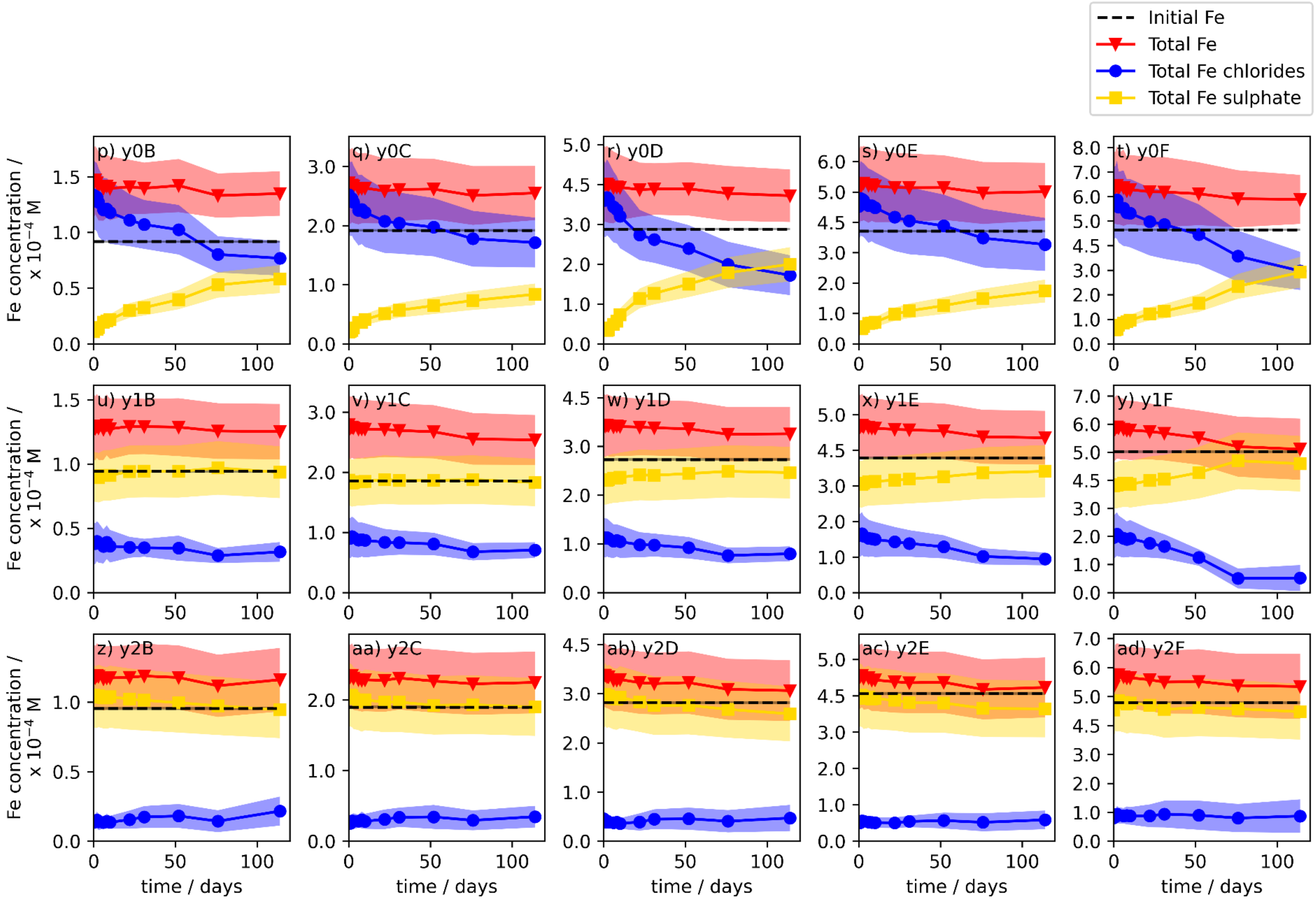
Initial Fe
Total Fe
Total Fe chlorides
Total Fe sulphate
p) y0B
q) y0C
r) y0D
s) y0E
t) y0F
u) y1B
v) y1C
w) y1D
x) y1E
y) y1F
z) y2B
aa) y2C
ab) y2D
ac) y2E
ad) y2F
Fe concentration / x 10^{-4} M
time / days

Figure S4: Fitted concentration of iron species in sample spectra for sample set x (a – o) and set y (p – ad) calculated by the non-linear fitting algorithm (main paper, Section 2.3). Shaded areas are a combination of the uncertainty in the components and the average uncertainty in that component molar absorptivity over the range with good signal-to-noise ratio (200 – 500 nm for chloride spectra, 200 – 450 nm for ferric sulphate spectra. These uncertainties are still underestimates as the fitted concentration of each species will be affected not only by the uncertainty in the absorptivity of that species, but by all species. With the exception of x D samples (c, h, m), which show a systematic error in the initial concentration of ferric chloride, the samples are generally within or just outside the initial concentrations of the solutions. The combination of the underestimated error due to inability to include uncertainties in molar absorptivities in the fitting programme, and the simple model that does not permit the formation of iron chloride-sulphate complexes accounts for the samples that are not within experimental uncertainty of the initial concentrations.

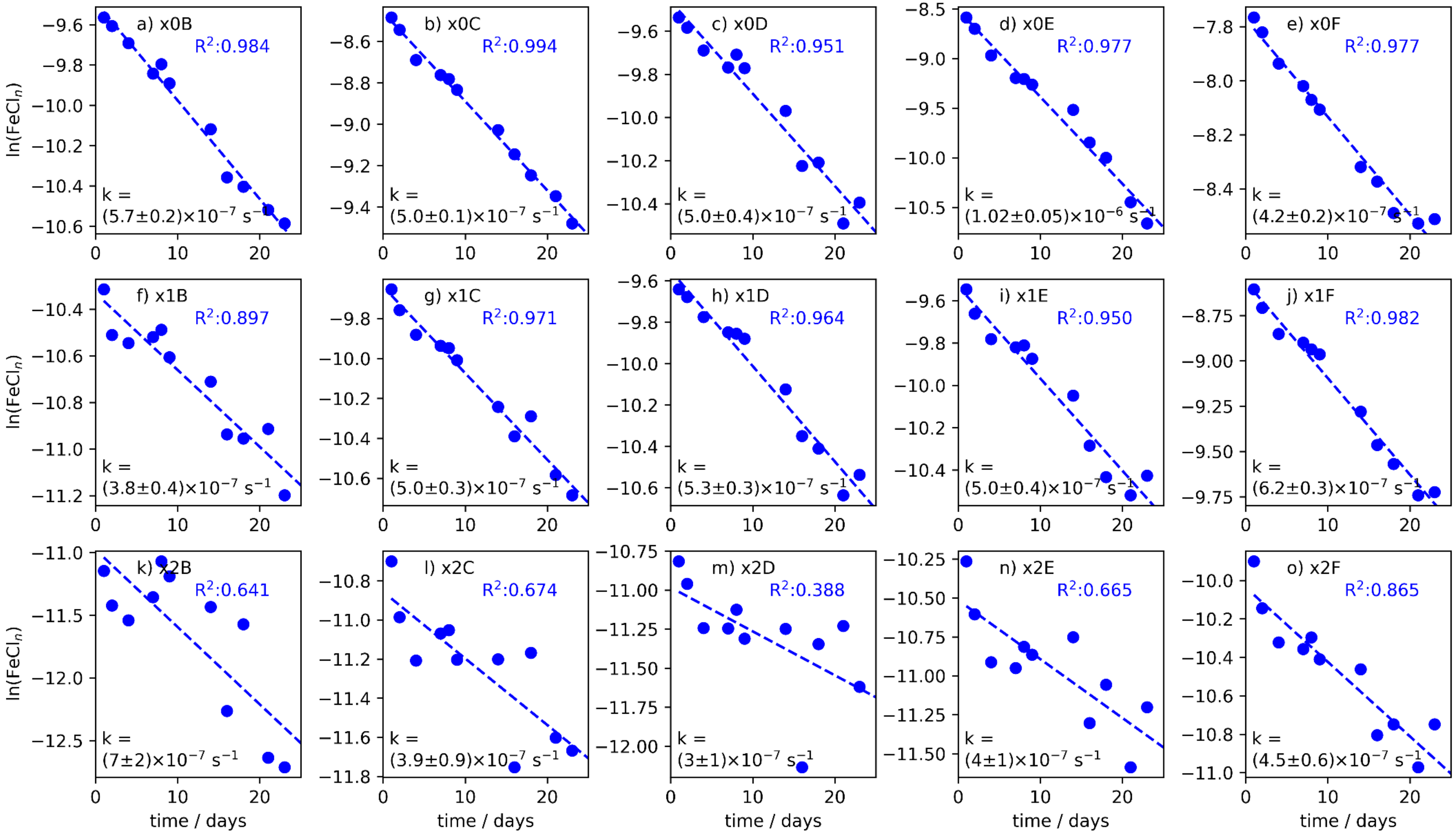

a) x0B
R2:0.984
k = (5.7±0.2)×10−7 s−1
b) x0C
R2:0.994
k = (5.0±0.1)×10−7 s−1
c) x0D
R2:0.951
k = (5.0±0.4)×10−7 s−1
d) x0E
R2:0.977
k = (1.02±0.05)×10−6 s−1
e) x0F
R2:0.977
k = (4.2±0.2)×10−7 s−1
f) x1B
R2:0.897
k = (3.8±0.4)×10−7 s−1
g) x1C
R2:0.971
k = (5.0±0.3)×10−7 s−1
h) x1D
R2:0.964
k = (5.3±0.3)×10−7 s−1
i) x1E
R2:0.950
k = (5.0±0.4)×10−7 s−1
j) x1F
R2:0.982
k = (6.2±0.3)×10−7 s−1
k) x2B
R2:0.641
k = (7±2)×10−7 s−1
l) x2C
R2:0.674
k = (3.9±0.9)×10−7 s−1
m) x2D
R2:0.388
k = (3±1)×10−7 s−1
n) x2E
R2:0.665
k = (4±1)×10−7 s−1
o) x2F
R2:0.865
k = (4.5±0.6)×10−7 s−1
ln(FeCln)
time / days

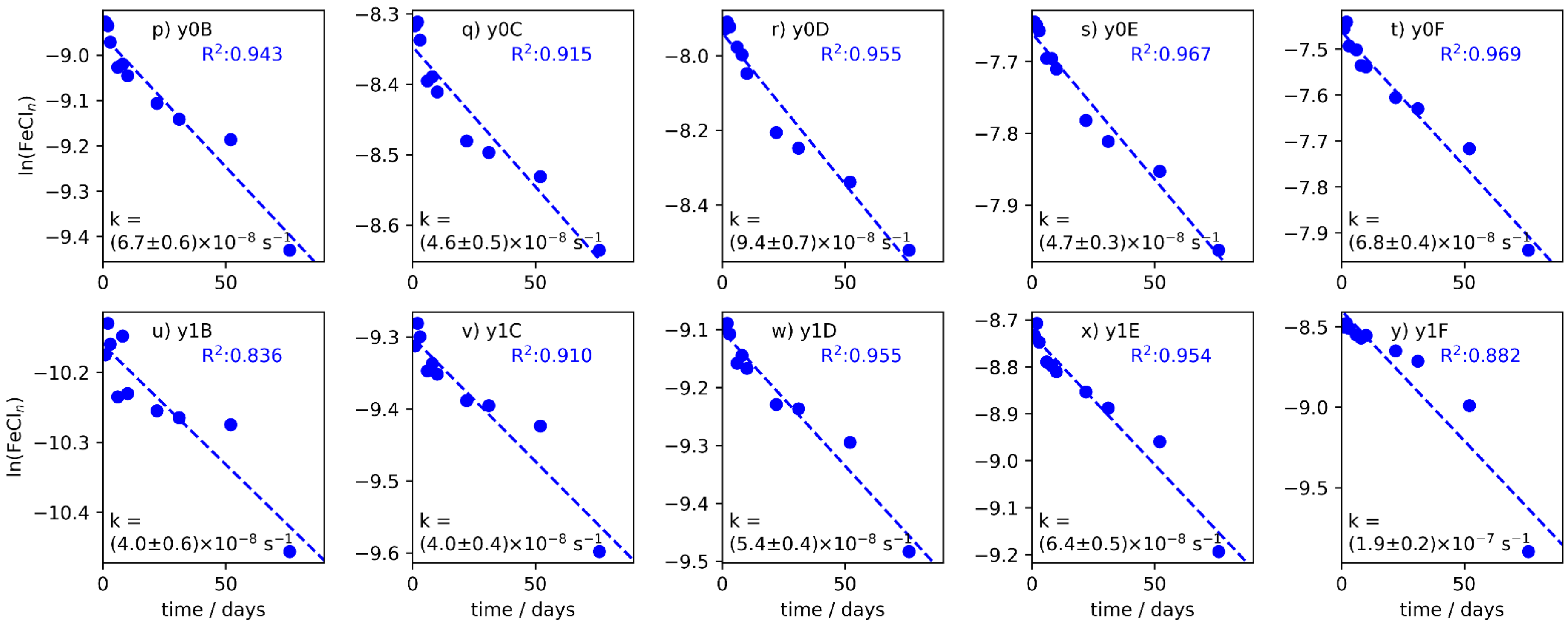

Figure S5. Linear fit for samples assuming 1st-order kinetics. The calculated pseudo-first order rate constant for each sample is shown on the graph. y2 samples showed no measurable change in fitted chloride concentration with time and the rate could not be estimated.

S3. Theoretical absorption spectra of $FeCl_3$ solvated in H_2O and H_2SO_4

Electronic structure calculations were performed using the B3LYP functional with the 6-311+g(2d,p) basis set [1]. The $FeCl_3$ molecule was placed in a cavity within the solvent reaction field, using the Polarizable Continuum Model [2]. For H_2O, the static and dynamic dielectric constants were set to $\varepsilon = 78.4$ and $\varepsilon_\infty = 1.77$ [3], respectively. In the case of H_2SO_4, these were set to $\varepsilon = 101.0$ [4] and $\varepsilon_\infty = 2.01$ [3]. The $FeCl_3$ geometry was first optimized in each of the solvent cavities, and the optical absorption cross sections were then calculated using time-dependent density function theory at the TD//B3LYP/6-311+g(2d,p) level of theory (for the first 30 excited states) [1]. The resulting absorption cross-sections are plotted as a function of wavelength in Figure S6. As iron (in particular) and chlorine are heavy atoms, and $FeCl_3$ is in a state of high (quintet) spin multiplicity, the calculated cross-sections are not expected to reproduce the experimental spectra very accurately in terms of the absorption peak, but the two calculated spectra can be reliably compared with one another. Comparison of the spectra in pure H_2SO_4 and pure H_2O reveals only small changes (less than 7.2%) in the absolute absorption cross section, indicating that the effect of replacing H_2SO_4 by H_2O ligands is modest. Hence, the effect of greater water availability in regions of lower acidity in the cloud should have a very small effect on the validity of using the measured spectra to model the Venusian atmospheric absorption.

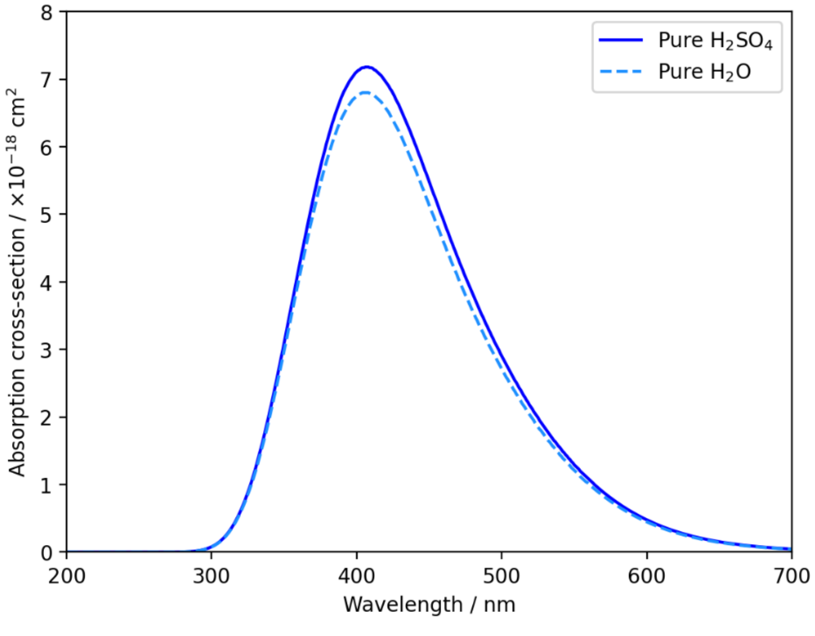

Figure S6. Theoretical absorption spectra of $FeCl_3$ solvated in either H_2O or H_2SO_4, calculated using at the TD-B3LYP level of theory with the Polarizable Continuum Model.

References

1. Frisch, M.J., et al., *Gaussian 16 Rev. B.01*. 2016, Wallingford, CT: Gaussian Inc.
2. Lipparini, F., et al., *A variational formulation of the polarizable continuum model.* J. Chem. Phys., 2010. **133**(1).

3. Lide, D.R., *Handbook of Physics and Chemistry*. Vol. 87th. edn. 2006, Boca Raton, FL: CRC Press.
4. Gillespie, R.J. and R.H. Cole, *The dielectric constant of sulphuric acid.* Trans. Faraday Soc., 1956. **52**: p. 1325-1331
